# Assessing the barriers and enablers to the implementation of the diagnostic radiographer musculoskeletal X-ray reporting service within the NHS in England: a systematic literature review

**DOI:** 10.1186/s12913-023-10161-y

**Published:** 2023-11-16

**Authors:** P. Lockwood, C. Burton, N. Woznitza, T. Shaw

**Affiliations:** 1 Present address: School of Allied Health Professions, Faculty of Medicine, Health and Social Care, Canterbury Christ Church University, North Holmes Road, Canterbury, Kent UK; 2 Radiology Department, University College London Hospitals NHS Foundation Trust, 235 Euston Road, London, UK

**Keywords:** Diagnostic radiographer, Reporting radiographer, X-rays, Musculoskeletal, Implementation, Enabler, Barrier

## Abstract

**Introduction:**

The United Kingdom (UK) government's healthcare policy in the early 1990s paved the way adoption of the skills mix development and implementation of diagnostic radiographers' X-ray reporting service. Current clinical practice within the public UK healthcare system reflects the same pressures of increased demand in patient imaging and limited capacity of the reporting workforce (radiographers and radiologists) as in the 1990s. This study aimed to identify, define and assess the longitudinal macro, meso, and micro barriers and enablers to the implementation of the diagnostic radiographer musculoskeletal X-ray reporting service in the National Healthcare System (NHS) in England.

**Methods:**

Multiple independent databases were searched, including PubMed, Ovid MEDLINE; Embase; CINAHL, and Google Scholar, as well as journal databases (Scopus, Wiley), healthcare databases (NHS Evidence Database; Cochrane Library) and grey literature databases (OpenGrey, GreyNet International, and the British Library EthOS depository) and recorded in a PRISMA flow chart. A combination of keywords, Boolean logic, truncation, parentheses and wildcards with inclusion/exclusion criteria and a time frame of 1995–2022 was applied. The literature was assessed against Joanna Briggs Institute's critical appraisal checklists. With meta-aggregation to synthesize each paper, and coded using NVivo, with context grouped into macro, meso, and micro-level sources and categorised into subgroups of enablers and barriers.

**Results:**

The wide and diverse range of data (*n* = 241 papers) identified barriers and enablers of implementation, which were categorised into measures of macro, meso, and micro levels, and thematic categories of context, culture, environment, and leadership.

**Conclusion:**

The literature since 1995 has reframed the debates on implementation of the radiographer reporting role and has been instrumental in shaping clinical practice. There has been clear influence upon both meso (professional body) and macro-level (governmental/health service) policies and guidance, that have shaped change at micro-level NHS Trust organisations. There is evidence of a shift in culturally intrenched legacy perspectives within and between different meso-level professional bodies around skills mix acceptance and role boundaries. This has helped shape capacity building of the reporting workforce. All of which have contributed to conceptual understandings of the skills mix workforce within modern radiology services.

**Supplementary Information:**

The online version contains supplementary material available at 10.1186/s12913-023-10161-y.

## Background

The implementation of the diagnostic radiographer musculoskeletal X-ray reporting service within National Healthcare System (NHS) clinical practice in England is now an established advanced clinical practice role. Although, the progression of increasing the radiographer reporting workforce has been slow. As a profession, radiography in England officially originated in 1920 with the formation of the Society of Radiographers (SoR), establishing qualifications and standards of practice, of which reporting of X-ray examinations for diagnosis was commonplace by non-medical radiographers, laypersons [1–3] and soldiers in late nineteenth century military campaigns [4] and the first world war [1–3, 5–13]. Between 1923 and 1925, the General Medical Council (GMC) and the British Medical Association (BMA) pressured a resolution to Articles 27 and 28 of the SoR Articles of Association to legally prevent radiographers from providing reports and diagnoses from X-ray examinations to protect the newly emerging medical profession of radiologists (previously termed medical-radiographers) [1, 5, 12, 13].

Although the discussion on radiographers reporting and diagnosing was raised further in 1929 [1] with the affiliation of the SoR and the British Institute of Radiology (BIR), and in 1975 [14, 15] in response to workforce shortages and reporting workload increases [16]. It wasn't until 1977 that the College of Radiographers (CoR) was formed to oversee education and professional responsibility (forming the joint Society and College of Radiographers (SCoR) professional body). The CoR amended Article 21 of the 'Articles of Association for Radiographers' in 1978 [17] to legally allow diagnostic radiographers to report Ultrasound (US) examinations. This critical and consequential shift in the scope of radiographers' practice was supported further by the Forrest Report [18] recommendations on mammography reporting and the concept of Red Dot [19] practice pressured the CoR 'Code of Professional Conduct' [20] to include "*a radiographer may provide a description of images, measurements and numerical data*"(1988, p.4).

The NHS drive for patient-focused improvements in England through White Paper policy reform such as 'Health of the Nation' [21], and delays to reporting [22], prompted pilot trials of X-ray reporting by radiographers by Saxton [23], Chapman [24], Loughran [25, 26], and Wilson [27]. The CoR supported in partnership and in combined working groups with the Department of Health (DoH), and the Royal College of Radiologists (RCR), moved to amend of the CoR 'Code of Professional Conduct' [28], to allow radiographers to provide both verbal and written reports on images. The following year, the Audit Commission Report [29] evidenced backlogs in reporting due to the limited radiologist workforce impacting reporting delays and recommended the DoH commission work on training radiographers to interpret and report images. In response, the CoR accredited the first postgraduate reporting programmes in musculoskeletal X-ray for radiographers [30].

Since the development of radiographer musculoskeletal X-ray reporting in 1994, there has been a growing body of research supporting this scope of practice following radiology-based hierarchical efficacy frameworks [31–36]. Reviewing the technical efficacy of radiographers' training accuracy in reporting musculoskeletal X-ray images under exam conditions with robust reference standards in controlled conditions (diagnostic accuracy [37]) of observer performance studies [38–40]. With progression onto the clinical validity of radiographers' accuracy in reporting musculoskeletal X-ray images in clinical practice environments (diagnostic performance [37]) [26, 39, 41–47] and when compared against other healthcare professions' performance (diagnostic outcome [37]) [39, 46, 48–52]. Thereafter assessing the clinical utility of radiographer's musculoskeletal X-ray reports on the effect on diagnostic thinking efficacy (discharging of patients [53–57]), the therapeutic efficacy in aiding treatment, management and outcomes [54], and the societal efficacy of cost–benefit [58, 59].

Current NHS clinical practice reflects the same pressures as in the 1990s. Implementation of musculoskeletal X-ray reporting by radiographers by the NHS and stakeholders has been slow to adjust and adapt whilst population growth has accelerated, evidenced in the continued backlog of reporting delays [60]. This study aimed to identify, define and assess the longitudinal macro, meso, and micro barriers and enablers to the implementation of the diagnostic radiographer musculoskeletal X-ray reporting service in the NHS in England.

## Methods

The protocol for this systematic review was registered with the International Prospective Register of Systematic Reviews (PROSPERO, registration number: CRD42022384191) and follows a predetermined published protocol in accordance with the reporting guidance provided in the Preferred Reporting Items for Systematic Reviews and Meta-Analysis Protocols (PRISMA-P) statement [61] (Additional files 1, 2).

### Study search strategy

The PICOs [62, 63] (Population, Intervention, Comparison, Outcomes study design) framework was used to structure the search strategy. Search terms combined keywords using operators (AND/OR) and Boolean logic to connect words, phrases, and similar concepts (synonyms), with the use of truncation, parentheses, and wildcards (Table 1).

**Table 1:** Search terms

Key search terms
“Radiographer X-ray reporting*” and/or “Diagnostic Radiographer X-ray reporting” and/or “Reporting Radiographer service” and/or “radiographer medical image reporting*” and/or “enablers*” and/or “drivers*” and/or “facilitators*” and/or “implementation*”
“Radiographer X-ray reporting*” and/or “Diagnostic Radiographer X-ray reporting” and/or “Reporting Radiographer service” and/or “radiographer medical image reporting*” and/or “barriers*” and/or “opposition*” and/or “restrictions*”

### Participants/population characteristics

Literature reporting the implementation of diagnostic radiographers reporting musculoskeletal X-ray examinations in the NHS in England.

### Intervention characteristics

The experimental intervention was classed as the musculoskeletal X-ray radiographer X-ray reporting service in the NHS in England. The controlled intervention was the existing consultant radiologist musculoskeletal X-ray reporting service in the NHS in England.

### Comparators

There was no comparator assessment of data against the consultant radiologist role or service, other than what was reported in the literature from observer performance studies.

### Outcomes

The primary outcome measures were to identify, define and assess against a socio-institutional theoretical model of macro, meso, and micro-levels [64] (Table 2) of enablers and barriers to the implementation of diagnostic radiographers reporting musculoskeletal X-ray examinations in the NHS in England since 1995.

**Table 2:** The subdivided systems levels for the contextual analysis

Macro	Meso	Micro
National governmental level such as: NHS/HEE/CQC	Professional body level: such as RCR, SCoR, BIR	Local frontline level such as: Individual sites, hospitals, universities

Multiple electronic databases were searched in January 2023, including PubMed, Ovid MEDLINE; Ovid Embase; CINAHL, and Google Scholar, as well as journal databases (ScienceDirect, Wiley), healthcare databases (NHS Knowledge and Library Hub Database; Cochrane Library) and grey literature databases (OpenGrey, GreyNet International, and the British Library EthOS depository).

### Inclusion and eligibility criteria

Published peer-review articles that discuss or identify the enablers or barriers to the reporting radiographer service in England including grey literature (such as reports, thesis, research, technical papers, conference papers, government documents, white papers, and evaluations). Defined by the 'Luxembourg Convention' definition [65] as (grey) literature produced on all levels of government, academics, business and industry, in print and electronic formats that discuss or identify the subject topic was reviewed, whilst identifying where bias may be present and the level of empirical evidence found within the grey literature. The exclusion criteria included non-english language papers, studies based on radiographic practice outside of England or private healthcare settings, and diagnostic imaging modalities other than X-ray (Table 3).

**Table 3:** Inclusion and exclusion criteria

Inclusion	Exclusion
Published post 1995	Pre 1995
English language	Non-English language
Peer-review articles	Duplicate literature
Grey literature	Private healthcare sector
Books and Documents	Non-English radiology departments
Case Reports	Non-reporting radiographer roles
Clinical Studies/trials	Therapeutic radiography
Commentary	Chest X-ray
Editorial	Abdomen X-ray
Government Publications	Mammography/Breast imaging
Guidelines	Non-X-ray imaging modalities
Historical articles	Commenting studies
Meta-Analysis	Red Dot studies
Systematic Reviews	Visual Perception / Eye tracking
Radiology within England	Preliminary Clinical Evaluation studies
Reporting radiographer service	Inter/Intra reader variables studies
NHS service provision	Errors/Bias in image interpretation
Diagnostic radiography	Chest/Abdomen X-ray
X-ray imaging	AI Image Interpretation/reporting
Musculoskeletal X-ray	Machine Learning Interpretation/reporting
	Veterinary Reporting
	Pathology Case Reports

### Screening

Screening and data extraction was performed with Rayyan [66] software with the assistance of a reference management tool [67]. The inclusion period started from 1995 when the first diagnostic radiographers graduated from an SCoR validated postgraduate clinical reporting programme with a qualification to report musculoskeletal X-rays. Literature from this period will discuss the implementation and facilitation of the role (practically). Although there are many papers pre-1995 that discuss the potential for the role (theoretically) and argue the need for clinical practice development and scope of practice, these papers do not discuss the practical enablers and barriers of the implemented role in practice.

The title, abstract and keywords were evaluated to determine each article for inclusion. If there was uncorrelated information in the title and abstract to determine inclusion, the full paper was retrieved and reviewed to resolve and determine the decision. Studies were excluded based on unrelated titles, abstract and full-text reviews, or duplication with a record documenting the reasoning.

### Data extraction and analysis

Data were extracted from the selected studies (Table 4). To address the wide and diverse range of data found, the results were analysed against the Joanna Briggs Institute [68] (JBI) validated critical appraisal checklists (Table 5) for validity, transparency, and rigor. The findings were displayed in a thematic matrix, and a meta-aggregation [69, 70] (different methodologies in the found literature) of the qualitative data into categories (macro, meso, and micro), and synthesize the findings into subthemes. A meta-analysis was not performed as the aim of this paper was not a quantitative summary of observer performance (efficacy) to justify the role, or against a comparator group (improve the power of a study or answer a hypothesis). Additionally, it was expected the various found observer performance quantitative data would contain significant heterogeneity within the different sample sizes, conduct, statistical analysis, and effect sizes.

**Table 4:** Data collection criteria

Data screening and extraction
Source full reference
Author/Institute
Year of literature
Country
Classification of literature
Main topic area
Summary of literature
Macro, meso, micro level
Enabler or barrier
Critical appraisal review score

**Table 5:** JBI critical appraisal tools

JBI Critical appraisal tools
Checklist for Analytical Cross Sectional Studies
Checklist for Case Control Studies
Checklist for Case Reports
Checklist for Case Series
Checklist for Cohort Studies
Checklist for Diagnostic Test Accuracy Studies
Checklist for Economic Evaluations
Checklist for Prevalence Studies
Checklist for Qualitative Research
Checklist for Quasi-Experimental Studies
Checklist for Randomized Controlled Trials
Checklist for Systematic Reviews
Checklist for Text and Opinion

The meta-aggregation [69, 70] was completed to synthesize each paper, with the findings coded using NVivo [71]. The context was grouped into macro, meso, and micro-level [64] sources and then categorised into subgroups of enablers and barriers. The results were displayed in a PRISMA [72] flow chart, with the findings displayed in a thematic matrix (in historical context ordering) with the subthemes and JBI [68] outcome scores. The search was conducted by the principal author, to minimise selection bias, all selected papers and results were checked by the researchers, two having radiography backgrounds (knowledge of healthcare research and the topic) and two having nursing backgrounds (knowledge of healthcare research and policy). If differences between researchers on the included literature occurred, a consensus final decision approach was agreed.

## Results

**Fig. 1:**
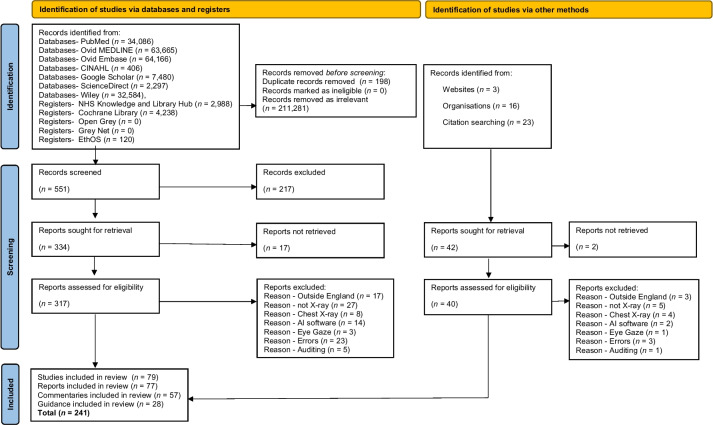
PRISMA flowchart results

There were limitations as to any literature search due to some databases (PubMed, Ovid Medline, Ovid Embase) not having the capacity to filter studies based on context/topic such as '*diagnostic radiography'* or *'X-ray imaging'* or geographic location (*England*). Database search results focused predominantly on phrases such as '*enablers, drivers, barriers, facilitators, implementation, opposition, and restrictions*'. However, database results were dominated by papers that included these keywords but contained irrelevant subjects that did not meet the inclusion criteria. In total, *n* = 241 papers were included in the results (Fig. 1) and displayed in thematic matric (Table 6). Covering a range of literature from observational studies (*n* = 16), surveys (*n* = 25), randomised control trials (*n* = 5), case studies (*n* = 8), literature reviews (*n* = 17), economic analysis (*n* = 1), clinical audits (*n* = 4), thesis (*n* = 3), book chapters (*n* = 2), governmental reports (*n* = 10), parliamentary reports (*n* = 3), NHS reports (*n* = 35), workforce reports (*n* = 22), professional body guidance documents (*n* = 28) reports (*n* = 7), and statements (*n* = 8), and expert commentaries (*n* = 47).

**Table 6:** List of articles for data extraction under macro, meso, micro levels, barriersˣ and enablers^◊^ subthemes, and JBI appraisal scores (cross-sectional studies*; Case Controlled Studies**; Case study***; Cohort Study****; Diagnostic Test Accuracy Studies^†^; Economic Evaluation^††^; Qualitative Research^‡^; RCTs^‡‡^; Systematic Reviews^§^; Text and Opinion^§§^)

Author(s)	Month/Year	Literature Category	Macro level		Meso level	Micro level	Workforce shortageˣ	Opposition to delegation of tasksˣ	Clinical management supportˣ	Training and educationˣ	Increasing patient demand◊	Increasing the workforce◊
Audit Commission [29]	Jan 1995	Govt. Report	**●**				**●**				**●**	
Wilson [27]	Jun 1995	Observational Study				**●**	**●**					
Brady [73]	Oct 1995	Commentary				**●**		**●**				
Paterson [74]	Oct 1995	Survey				**●**						
College of Radiographers [75]	Oct 1995	Guidance			**●**							
Royal College of Radiologists [76]	Oct 1995	Statement			**●**			**●**	**●**			
Loughran [26]	Feb 1996	Observational Study				**●**						
Field-Boden, Piper [77]	Mar 1996	Commentary				**●**	**●**					
Royal College of Radiologists [78]	July 1996	Statement			**●**				**●**			
Field-Boden, Piper [79]	Sept 1996	Commentary				**●**						
Williams [80]	Oct 1996	Commentary				**●**			**●**			
Brindle [81]	Nov 1996	Commentary				**●**	**●**	**●**	**●**	**●**		
Kletzenbauer [82]	Nov 1996	Survey				**●**						
Robinson [42]	Dec 1996	Observational Study				**●**						
Chapman [83]	Aug 1997	Commentary				**●**	**●**			**●**		
Cunningham [84]	Sept 1997	Commentary				**●**	**●**					
College of Radiographers [85]	Oct 1997	Guidance			**●**							
Eyres, et al. [86]	Dec 1997	Survey				**●**	**●**					
Department of Health [87]	Jul 1998	NHS Report	**●**				**●**					
Robinson [88]	Aug 1998	Commentary				**●**			**●**	**●**		
College of Radiographers [89]	Aug 1998	Guidance			**●**		**●**					
Piper, Paterson, Ryan [90]	Mar 1999	Observational Study				**●**						
Prime, Paterson, Henderson [91]	May 1999	Survey				**●**						
Carter, Manning [40]	May 1999	Case Study				**●**						
Robinson, Culpan, Wiggins [41]	June 1999	Audit				**●**						
Fernando [92]	Aug 1999	Commentary				**●**	**●**					
Royal College of Radiologists [93]	Aug 1999	Guidance	**●**				**●**			**●**	**●**	
Price, Miller, Payne [94]	Feb 2000	Survey				**●**						
Beecham [95]	Apr 2000	Commentary				**●**						
Department of Health [96]	Apr 2000	NHS Report	**●**									
Department of Health [97]	July 2000	NHS Report	**●**								●	●
NHS England [98]	July 2000	NHS Report	**●**									
Tennant [99]	Aug 2000	Commentary				**●**	●	●				
Department of Health [100]	Sept 2000	NHS Report	**●**									●
Price [12]	Oct 2000	Commentary				**●**	●	●	●	●		
Department of Health [101]	Nov 2000	NHS Report	**●**								●	●
Brayley [102]	Nov 2000	Commentary				**●**	**●**	**●**				**●**
Nixon [103]	Feb 2001	Commentary				**●**						
Price [104]	May 2001	Commentary				**●**	●	●	●	●		
Brealey [105]	May 2001	Commentary				**●**						●
Brealey [106]	Nov 2001	Commentary				**●**						
Brealey [107]	Feb 2002	Survey				**●**		**●**				
Royal College of Radiologists [108]	Apr 2002	Workforce Report			**●**		●	●	●	●	●	
Price, Paterson [109]	May 2002	Commentary				**●**						
Hayes [110]	Jun 2002	Commentary				**●**						
Price, Miller, Mellor [111]	Nov 2002	Survey				**●**	●				●	
Brealey, Scally, Thomas [112]	Nov 2002	Literature Review				**●**						●
Morris, et al. [113]	Nov 2002	Commentary				**●**						
Reed [114]	Dec 2002	Commentary				**●**	**●**	**●**		**●**	**●**	
NHS England [115]	Jan 2003	NHS Report	**●**									
Brealey, et al. [48]	Jan 2003	RCT				**●**	●				●	●
Rudd [116]	Feb 2003	Commentary				**●**	●	●				
College of Radiographers [117]	Apr 2003	Guidance			**●**		●	●				
Department of Health [118]	Jun 2003	Report/Case studies	**●**									●
Alderson, Hogg [119]	Nov 2003	Commentary				**●**						
Brealey [120]	Jan 2004	Thesis				**●**	**●**	**●**			**●**	**●**
House of Commons [121]	Jun 2004	Govt. Report	**●**									
Department of Health [122]	Aug 2004	NHS Report				**●**		●				
Paterson, et al. [123]	Aug 2004	Commentary				**●**						
Reeves [124]	Aug 2004	Case Study				**●**						
House of Commons [125]	Oct 2004	Govt. Report	**●**				●				●	●
Brealey, et al. [43]	Feb 2005	Literature Review				**●**	**●**				**●**	
Piper, Paterson, Godfrey [38]	Feb 2005	Observational Study				**●**					●	●
House of Commons [126]	Mar 2005	Govt. Report	**●**				●				●	●
Brealey, et al. [58]	Jun 2005	Economic Analysis				**●**	●				●	●
Brealey, Scuffham [45]	Jun 2005	Observational Study				**●**	●				●	
Brealey, et al. [49]	Jun 2005	Observational Study				**●**	●				●	
Jones [127]	Jun 2005	Commentary				**●**					●	●
Dimond [128]	Jul 2005	Book Chapter				**●**						
College of Radiographers [129]	Oct 2005	Guidance			**●**		●					
Radovanovic, Armfield [130]	Dec 2005	Literature Review				**●**						
House of Commons [131]	Dec 2005	Govt. Report	**●**				●					●
Royal College of Radiologists [132]	Jan 2006	Guidance			**●**			●	●	●		
Donovan, Manning [133]	Feb 2006	Commentary				**●**	●	●		●		
House of Commons [134]	Mar 2006	Govt. Report	**●**				●					●
House of Commons [135]	Mar 2006	Govt. Report	**●**				●					●
Government Select Committee [136]	Mar 2006	Parliamentary Report	**●**				●	●	●	●	●	
Department of Health [137]	Mar 2006	NHS Report	**●**				●					●
Price [13]	Oct 2006	Thesis				**●**	●	●	●	●	●	●
College of Radiographers [138]	Oct 2006	Guidance			**●**		●	●			●	
Hardy, Snaith [139]	Nov 2006	Commentary				**●**						
Woodford [140]	Nov 2006	Literature Review				**●**	●				●	
Woolford, Hewitt [141]	Dec 2006	Commentary				**●**						
Royal College of Radiologists [142]	Jan 2007	Guidance			**●**		●				●	
Snaith [53]	Feb 2007	Observational Study				**●**						
NHS England [143]	Feb 2007	NHS Report	**●**									●
Price, Le Masurier [144]	Feb 2007	Commentary				**●**	●					●
House of Commons [145]	Mar 2007	Govt. Report	**●**				●					●
Snaith, Hardy [146]	May 2007	Commentary				**●**						
Smith, Baird [147]	Jun 2007	Commentary				**●**	●	●			●	
Humphreys, et al. [148]	Sept 2007	Literature Review				**●**						
Royal College of Radiologists [149]	Sept 2007	Guidance			**●**							
Blakeley, Hogg, Heywood [150]	Feb 2008	Observational Study				**●**						
Hardy, Snaith, Smith [151]	Apr 2008	Survey				**●**	●				●	●
Jones, Manning [152]	Aug 2008	Survey				**●**					●	
Price, et al. [153]	Nov 2008	Survey			**●**			●	●		●	●
Hardy, Spencer, Snaith [47]	Nov 2008	Observational Study			**●**							
Cowling [154]	Dec 2008	Commentary				**●**						
Hardy, et al. [155]	Dec 2008	Commentary				**●**	●			●		
Hogg, Hogg, Henwood [156]	Dec 2008	Commentary				**●**						
Buttress, Marangon [157]	Dec 2008	Commentary				**●**						
Kelly, Piper, Nightingale [158]	Dec 2008	Literature Review				**●**	●	●		●	●	
Hardy, Snaith [159]	May 2009	Survey				**●**						●
Health Professionals Council [160]	May 2009	Prof. Body Report	**●**									
McGee [161]	Jun 2009	Book Chapter				**●**						
Brealey, et al. [44]	Jul 2009	Literature Review				**●**	●					
College of Radiographers [162]	July 2009	Guidance			**●**			●	●	●		
Coleman, Piper [50]	Aug 2009	Observational Study				**●**						
Yielder, Davis [163]	Nov 2009	Commentary				**●**			●			
Smith, Reeves [164]	Dec 2009	Literature Review				**●**	●	●				●
College of Radiographers [165]	Mar 2010	Guidance			**●**							
Royal College of Radiologists [166]	Apr 2010	Guidance			**●**			●	●	●		
College of Radiographers [166]	May 2010	Guidance			**●**			●		●		
Paterson [167]	Jun 2010	Commentary				**●**		●		●		●
Department of Health [168]	July 2010	NHS Report	**●**									●
Price [169]	Aug 2010	Commentary				**●**		●				
Forsyth, Maehle [170]	Nov 2010	Survey				**●**						
Society of Radiographers [171]	Nov 2010	Commentary			**●**							●
Department of Health [172]	Jan 2011	NHS Report	**●**									
Royal College of Radiologists [173]	Apr 2011	Guidance			**●**			**●**	**●**	**●**		
Miller, Price, Vosper [174]	May 2011	Survey				**●**	**●**		**●**		**●**	
Hardy, Snaith [175]	Nov 2011	RCT				**●**	**●**				**●**	
House of Commons [176]	Dec 2011	Govt. Report	**●**				●				●	●
NHS England [177]	Jan 2012	NHS Report	**●**				●					●
Department of Health [178]	Jan 2012	NHS Report	**●**									**●**
Stephenson, et al. [179]	Apr 2012	Literature Review				**●**		**●**				
Society of Radiographers [180]	May 2012	Workforce Report			**●**							**●**
Royal College of Radiologists [181]	Jun 2012	Workforce Report			**●**		**●**	**●**		**●**	**●**	
Royal College of Radiologists [182]	Sept 2012	Guidance			**●**		**●**				**●**	
Royal College of Radiologists [183]	Sept 2012	Guidance			**●**							
Royal College of Radiologists [184]	Oct 2012	Guidance			**●**						**●**	
Centre for Workforce Intelligence [185]	Dec 2012	Workforce Report	**●**				●			●	●	●
Buskov, et al. [52]	Jan 2013	Observational Study				**●**	**●**				**●**	
Hardy, Snaith, Scally [54]	Jan 2013	RCT				**●**					**●**	
College of Radiographers [186]	Jan 2013	Guidance			**●**							
Field, Snaith [187]	Feb 2013	Commentary				**●**	**●**				**●**	
College of Radiographers [188]	Feb 2013	Guidance			**●**						**●**	**●**
Hardy, Hutton, Snaith [59]	Feb 2013	RCT				**●**					**●**	
Society of Radiographers [55, 189]	Mar 2013	Workforce Report				**●**						
Henderson, Gray, Booth [55]	Mar 2013	Clinical Audit				**●**						
Health and Care Professions Council [160]	May 2013	Guidance	**●**									
Leishman [190]	May 2013	Survey				**●**	**●**					
Snaith, Hardy [191]	May 2013	Survey				**●**						
Snaith, Hardy [56]	Apr 2014	RCT				**●**						
Woznitza [192]	May 2014	Commentary				**●**						
Cox, Price [193]	May 2014	Survey				**●**						
Woznitza, et al. [194]	Aug 2014	Clinical Audit				**●**	**●**				**●**	
Royal College of Radiologists [195]	Sept 2014	Prof. Body Report			**●**		**●**		**●**	**●**	**●**	
Piper [46]	Sept 2014	Thesis				**●**	**●**					**●**
Royal College of Radiologists [196]	Oct 2014	Workforce Report			**●**		**●**			**●**	**●**	
NHS England [197]	Oct 2014	NHS Report	**●**							**●**		
Society of Radiographers [198]	Nov 2014	Workforce Report			**●**		**●**					**●**
Royal College of Radiologists [199]	Jan 2015	Statement			**●**		**●**			**●**	**●**	
Snaith, Hardy, Lewis [200]	May 2015	Survey				**●**		**●**	**●**		**●**	
Barter [201]	July 2015	Literature Review				**●**		**●**				
Independent Cancer Taskforce [202]	July 2015	NHS Report	**●**				**●**				**●**	
Royal College of Radiologists [203]	July 2015	Workforce Report			**●**		**●**			**●**	**●**	
Royal College of Radiologists [204]	Dec 2015	Guidance			**●**			**●**				
Royal College of Radiologists [205]	Feb 2016	Statement			**●**		**●**	**●**	**●**		**●**	
Booth, Henwood, Miller [206]	Feb 2016	Case Study				**●**						
Hardy, et al. [207]	Apr 2016	Literature Review				**●**	**●**				**●**	
Royal College of Radiologists [208]	July 2016	Guidance			**●**					**●**		
Milner, Culpan, Snaith [209]	Aug 2016	Survey				**●**	**●**	**●**	**●**		**●**	
Nightingale, McNulty [210]	Aug 2016	Commentary				**●**	**●**					
Royal College of Radiologists [211]	Sept 2016	Workforce Report			**●**		**●**			**●**	**●**	
Snaith, Milner, Harris [212]	Nov 2016	Observational Study				**●**						
Royal College of Radiologists [213]	Nov 2016	Prof. Body Report			**●**		**●**		**●**		**●**	
Society of Radiographers [214]	Nov 2016	Workforce Report			**●**		**●**					**●**
NHS England [215]	Dec 2016	NHS Report		**●**			**●**				**●**	
NHS England [216]	Jan 2017	NHS Report		**●**			**●**					**●**
Milner, Snaith [217]	Feb 2017	Survey				**●**	**●**				**●**	
Lockwood [218]	Mar 2017	Survey				**●**			**●**			**●**
Benwell, Fowler [219]	Mar 2017	Survey				**●**		**●**	**●**			**●**
British Institute of Radiology [220]	May 2017	Prof. Body Report			**●**		**●**				**●**	**●**
Health Education England [221]	May 2017	NHS Report		**●**								**●**
Society of Radiographers [222]	May 2017	Statement			**●**							**●**
Society of Radiographers [223]	May 2017	Workforce Report			**●**		**●**				**●**	**●**
Synergy News [224]	Jun 2017	Commentary				**●**						**●**
Kerrney [225]	Aug 2017	Commentary				**●**	**●**	**●**	**●**			**●**
Royal College of Radiologists [226]	Oct 2017	Workforce Report			**●**		**●**				**●**	**●**
NHS England [227]	Dec 2017	NHS Report		**●**								**●**
NHS England [228]	Dec 2017	NHS Report		**●**			**●**				**●**	
Thom [229]	Feb 2018	Literature Review				**●**	**●**	**●**	**●**		**●**	
Synergy News [230]	Mar 2018	Commentary				**●**					**●**	**●**
Royal College of Radiologists [231]	Mar 2018	Guidance			**●**			**●**	**●**			
NHS England [232]	Apr 2018	NHS Report		**●**								
Care Quality Commission [233]	July 2018	NHS Report		**●**			**●**					**●**
Harcus, Snaith [234]	Sept 2018	Case Study				**●**						**●**
Royal College of Radiologists [235]	Sept 2018	Workforce Report			**●**		**●**				**●**	**●**
Society of Radiographers [236]	Oct 2018	Workforce Report			**●**							**●**
NHS England [237]	Jan 2019	NHS Report		**●**								
Snaith, et al. [238]	Jan 2019	Clinical Audit				**●**						
Royal College of Radiologists [239]	Apr 2019	Workforce Report			**●**		**●**				**●**	**●**
Culpan, et al. [240]	May 2019	Literature Review				**●**	**●**	**●**	**●**			**●**
Society of Radiographers [241]	May 2019	Workforce Report			**●**							**●**
Royal College of Radiologists [242]	May 2019	Prof. Body Report			**●**		**●**			**●**		**●**
Society of Radiographers [243]	Jun 2019	Statement			**●**							**●**
NHS England [244]	Jun 2019	NHS Report		**●**			**●**					**●**
Health Education England [245]	July 2029	NHS Report		**●**							**●**	**●**
NHS England [246]	July 2019	NHS Report		**●**							**●**	**●**
Health Education England [247]	Aug 2019	NHS Report		**●**							**●**	**●**
Health Education England [248]	Aug 2019	NHS Report		**●**								**●**
Stevens [249]	Aug 2019	Survey				**●**	**●**				**●**	
Cuthbertson [250]	Oct 2019	Phenomenological				**●**		**●**	**●**			**●**
Harcus, Snaith [251]	Nov 2019	Case Study				**●**					**●**	**●**
NHS England [252]	Nov 2019	NHS Report		**●**			**●**				**●**	**●**
Cuthbertson [253]	Feb 2020	Phenomenological				**●**		**●**	**●**		**●**	**●**
Royal College of Radiologists [254]	Apr 2020	Workforce Report			**●**		**●**				**●**	**●**
Society of Radiographers [255]	May 2020	Statement			**●**							
Society of Radiographers [256]	May 2020	Workforce Report			**●**		**●**					**●**
NHS England [257]	July 2020	NHS Report		**●**								**●**
Price, Paterson [6]	Aug 2020	Commentary				**●**		**●**	**●**			
Woznitza, et al. [258]	Aug 2020	Commentary				**●**						**●**
NHS England [259]	Oct 2020	NHS Report		**●**			**●**			**●**	**●**	**●**
NHS England [260]	Nov 2020	NHS Report		**●**			**●**		**●**			**●**
Milner, Barlow [261]	Feb 2021	Survey				**●**		**●**				
Woznitza, et al. [262]	Feb 2021	Survey				**●**						
Society of Radiographers [263]	Apr 2021	Workforce Report			**●**		**●**					**●**
Society of Radiographers [264]	Apr 2021	Statement			**●**		**●**					**●**
Royal College of Radiologists [265]	Apr 2021	Prof. Body Report			**●**		**●**				**●**	
Royal College of Radiologists [266]	Apr 2021	Workforce Report			**●**		**●**				**●**	**●**
Heales, Mills, Ladd [267]	Jun 2021	Commentary				**●**					**●**	
Society of Radiographers [268]	Jun 2021	Workforce Report			**●**							
Parliamentary Ombudsman [269]	July 2021	Parliamentary Report		**●**								
Health Education England [270]	July 2021	NHS Report		**●**								●
Akpan, Kitundu, Ekpo [271]	Sept 2021	Literature Review				**●**						
College of Radiographers [272]	Sept 2021	Guidance			**●**							
Government Select Committee [273]	Jan 2022	Parliamentary Report		**●**			**●**	**●**		**●**	**●**	**●**
Cain, et al. [51]	Jan 2022	Observational Study			**●**	**●**				**●**		**●**
Wood [274]	Feb 2022	Literature Review				**●**	**●**	**●**			**●**	**●**
Shepherd, Lourida, Meertens [57]	Apr 2022	Literature Review				**●**					**●**	
NHS England [275]	Apr 2022	NHS Report		**●**			**●**					**●**
Royal College of Radiologists [276]	May 2022	Workforce Report			**●**		**●**	**●**	**●**	**●**	**●**	
Royal College of Radiologists [277]	May 2022	Guidance			**●**							**●**
Health Education England [278]	July 2022	Guidance		**●**								**●**
Sevens, McGivern [279]	Aug 2022	Survey				**●**			**●**		**●**	**●**
College of Radiographers [280]	Aug 2022	Workforce Report				**●**	**●**					**●**
The Kings Fund [281]	Oct 2022	Report			**●**		**●**				**●**	**●**
Academy of Medical Royal Colleges [282]	Oct 2022	Guidance			**●**							
Murphy, Nightingale, Calder [283]	Nov 2022	Literature Review				**●**	**●**	**●**	**●**			
Murphy, Nightingale, Calder [284]	Nov 2022	Literature Review				**●**		**●**	**●**	**●**		**●**

Author(s)	Reducing reporting TATs◊	Increasing reporter capacity◊	Improve patient care/treatment◊	Promoting advanced practice◊	Skills mix team working◊	Professional accountability◊	Clinical efficacy (competency)◊	Validity (accuracy/agreement)◊	Clinical utility (treatment decisions)◊	Economic value◊	JBI Appraisal score
Audit Commission [29]	**●**		**●**								5/6^§§^
Wilson [27]	**●**	**●**	**●**								7/10**
Brady [73]				**●**		**●**	**●**			**●**	6/6^§§^
Paterson [74]		**●**	**●**	**●**		**●**					6/8*
College of Radiographers [75]	**●**	**●**	**●**	**●**		**●**			**●**		5/6^§§^
Royal College of Radiologists [76]			**●**			**●**					5/6^§§^
Loughran [26]	**●**		**●**	**●**			**●**	**●**			8/10^†^
Field-Boden, Piper [77]	**●**	**●**	**●**	**●**			**●**				5/6^§§^
Royal College of Radiologists [78]					**●**	**●**					5/6^§§^
Field-Boden, Piper [79]	**●**	**●**	**●**	**●**	**●**		**●**				5/6^§§^
Williams [80]			**●**	**●**	**●**	**●**					5/6^§§^
Brindle [81]		**●**			**●**	**●**					4/6^§§^
Kletzenbauer [82]					**●**	**●**					7/8*
Robinson [42]	**●**	**●**	**●**		**●**		**●**	**●**			9/10**
Chapman [83]				**●**	**●**	**●**				**●**	5/6^§§^
Cunningham [84]		**●**		**●**	**●**	**●**					5/6^§§^
College of Radiographers [85]		**●**		**●**	**●**	**●**					5/6^§§^
Eyres, et al. [86]	**●**	**●**		**●**	**●**		**●**				5/8*
Department of Health [87]			**●**		**●**	**●**					5/6^§§^
Robinson [88]				**●**	**●**			**●**			4/6^§§^
College of Radiographers [89]			**●**	**●**	**●**	**●**					5/6^§§^
Piper, Paterson, Ryan [90]	**●**	**●**		**●**	**●**	**●**	**●**	**●**			8/10***
Prime, Paterson, Henderson [91]		**●**		**●**			**●**	**●**			5/8*
Carter, Manning [40]				**●**			**●**	**●**			9/10***
Robinson, Culpan, Wiggins [41]			**●**	**●**			**●**	**●**			8/10***
Fernando [92]		**●**			**●**						5/6^§§^
Royal College of Radiologists [93]					**●**					**●**	4/6^§§^
Price, Miller, Payne [94]				**●**	**●**						5/8*
Beecham [95]			**●**		**●**						4/6^§§^
Department of Health [96]			●	●	●						4/6^§§^
Department of Health [97]	●		●	●	●						5/6^§§^
NHS England [98]	●		●								5/6^§§^
Tennant [99]		●		●	●						6/6^§§^
Department of Health [100]	●	●	●		●						5/6^§§^
Price [12]		●			●	●					6/6^§§^
Department of Health [101]			●	●	●						5/6^§§^
Brayley [102]	**●**	**●**	**●**	**●**	**●**	**●**	**●**		**●**		6/6^§§^
Nixon [103]				**●**	**●**	**●**					6/6^§§^
Price [104]	●	●	●	●	●	●	●	●	●		6/6^§§^
Brealey [105]				●	●	●	●	●	●	●	6/6^§§^
Brealey [106]			**●**	**●**	**●**	**●**	**●**	**●**	**●**		5/6^§§^
Brealey [107]	**●**	**●**		**●**	**●**		**●**			**●**	7/8*
Royal College of Radiologists [108]											5/6^§§^
Price, Paterson [109]			●	●		●					5/6^§§^
Hayes [110]	●		●	●	●						4/6^§§^
Price, Miller, Mellor [111]		●			●						6/8*
Brealey, Scally, Thomas [112]							●	●			9/11^§^
Morris, et al. [113]	●		●	●	●						5/6^§§^
Reed [114]				**●**	**●**		**●**	**●**			5/6^§§^
NHS England [115]			**●**	**●**	**●**						5/6^§§^
Brealey, et al. [48]	●			●	●		●	●			12/13^‡‡^
Rudd [116]				●	●		●	●			5/11^§^
College of Radiographers [117]		●		●	●						5/6^§§^
Department of Health [118]	●			●	●	●					6/6^§§^
Alderson, Hogg [119]			**●**			**●**					6/6^§§^
Brealey [120]	**●**	**●**	**●**	**●**	**●**	**●**	**●**	**●**	**●**	**●**	10/10**
House of Commons [121]	●		●	●	●						5/6^§§^
Department of Health [122]			●	●	●		●	●		●	5/6^§§^
Paterson, et al. [123]	●	●	●	●		●	●	●	●	●	6/6^§§^
Reeves [124]				●			●	●			5/6^§§^
House of Commons [125]			●								5/6^§§^
Brealey, et al. [43]				**●**	**●**		**●**	**●**	**●**	**●**	11/11^§^
Piper, Paterson, Godfrey [38]		●		●			●	●			10/11****
House of Commons [126]			●								5/6^§§^
Brealey, et al. [58]	●	●	●	●	●		●	●	●	●	11/11^††^
Brealey, Scuffham [45]	●			●	●		●	●			8/10**
Brealey, et al. [49]				●	●		●	●	●		10/10**
Jones [127]	●		●	●	●		●		●		4/6^§§^
Dimond [128]				●		●			●		5/6^§§^
College of Radiographers [129]			●	●							5/6^§§^
Radovanovic, Armfield [130]			●	●			●	●			3/11^§^
House of Commons [131]					●						4/6^§§^
Royal College of Radiologists [132]									●		5/6^§§^
Donovan, Manning [133]					●		●				6/6^§§^
House of Commons [134]					●						4/6^§§^
House of Commons [135]					●						4/6^§§^
Government Select Committee [136]					●						4/6^§§^
Department of Health [137]				●	●						4/6^§§^
Price [13]		●		●	●	●			●		8/8*
College of Radiographers [138]	●	●	●	●	●	●	●	●	●		5/6^§§^
Hardy, Snaith [139]				●		●					6/6^§§^
Woodford [140]	●	●			●						3/11^§^
Woolford, Hewitt [141]				●	●	●	●				5/6^§§^
Royal College of Radiologists [142]	●		●	●	●	●					5/6^§§^
Snaith [53]	●		●	●	●		●	●	●		10/10**
NHS England [143]	●				●	●					4/6^§§^
Price, Le Masurier [144]		●		●	●						6/8*
House of Commons [145]					●						5/6^§§^
Snaith, Hardy [146]				●		●			●		5/6^§§^
Smith, Baird [147]	●			●			●	●		●	5/6^§§^
Humphreys, et al. [148]			●	●	●	●					9/11^§^
Royal College of Radiologists [149]						●	●				5/6^§§^
Blakeley, Hogg, Heywood [150]	●		●	●			●	●			8/10**
Hardy, Snaith, Smith [151]		●		●			●	●			6/8*
Jones, Manning [152]				●		●	●	●			6/8*
Price, et al. [153]				●		●					6/8*
Hardy, Spencer, Snaith [47]	●			●			●	●			10/10**
Cowling [154]				●							4/6^§§^
Hardy, et al. [155]				●							5/6^§§^
Hogg, Hogg, Henwood [156]				●							5/6^§§^
Buttress, Marangon [157]				●	●	●					5/6^§§^
Kelly, Piper, Nightingale [158]	●		●	●	●		●	●			3/11^§^
Hardy, Snaith [159]		●		●							7/8*
Health Professionals Council [160]			●			●					4/6^§§^
McGee [161]			●	●	●	●					4/6^§§^
Brealey, et al. [44]							●	●			5/11^§^
College of Radiographers [162]			●	●	●						5/6^§§^
Coleman, Piper [50]					●		●	●			9/10**
Yielder, Davis [163]				●	●	●					6/6^§§^
Smith, Reeves [164]			●	●	●			●			9/11^§^
College of Radiographers [165]			●	●	●	●					5/6^§§^
Royal College of Radiologists [166]											4/6^§§^
College of Radiographers [166]	●			●		●	●		●		5/6^§§^
Paterson [167]	●				●		●	●			6/6^§§^
Department of Health [168]			●			●					5/6^§§^
Price [169]						●					6/6^§§^
Forsyth, Maehle [170]			●	●							7/8*
Society of Radiographers [171]	●	●	●				●				4/6^§§^
Department of Health [172]	●										5/6^§§^
Royal College of Radiologists [173]	**●**					**●**					5/6^§§^
Miller, Price, Vosper [174]		**●**		**●**	**●**						7/8*
Hardy, Snaith [175]	**●**	**●**	**●**	**●**	**●**		**●**	**●**	**●**	**●**	12/13^‡‡^
House of Commons [176]				●	●						4/6^§§^
NHS England [177]	●	●	●	●	●						5/6^§§^
Department of Health [178]				**●**	**●**						4/6^§§^
Stephenson, et al. [179]	**●**			**●**		**●**	**●**	**●**			6/11^§^
Society of Radiographers [180]				**●**							7/8*
Royal College of Radiologists [181]		**●**		**●**	**●**					**●**	8/8*
Royal College of Radiologists [182]	**●**	**●**		**●**	**●**	**●**	**●**		**●**		5/6^§§^
Royal College of Radiologists [183]	**●**		**●**			**●**			**●**		5/6^§§^
Royal College of Radiologists [184]					**●**						5/6^§§^
Centre for Workforce Intelligence [185]		●		●	●					**●**	8/8*
Buskov, et al. [52]				**●**			●	●			10/10**
Hardy, Snaith, Scally [54]	**●**		**●**	**●**	**●**		**●**	**●**	**●**		13/13^‡‡^
College of Radiographers [186]				**●**	**●**	**●**	**●**				5/6^§§^
Field, Snaith [187]	**●**		**●**	**●**	**●**	**●**	**●**				6/6^§§^
College of Radiographers [188]	**●**			**●**	**●**	**●**	**●**				5/6^§§^
Hardy, Hutton, Snaith [59]	**●**			**●**	**●**					**●**	13/13^‡‡^
Society of Radiographers [55, 189]			**●**			**●**					7/8*
Henderson, Gray, Booth [55]	**●**		**●**	**●**	**●**		**●**	**●**	**●**		9/11****
Health and Care Professions Council [160]			**●**			**●**	**●**				4/6^§§^
Leishman [190]		**●**	**●**	**●**							7/8*
Snaith, Hardy [191]	**●**		**●**	**●**					**●**		8/8*
Snaith, Hardy [56]			**●**	**●**	**●**		**●**				13/13^‡‡^
Woznitza [192]				**●**			**●**	**●**			5/6^§§^
Cox, Price [193]	**●**			**●**							7/8*
Woznitza, et al. [194]	**●**		**●**	**●**	**●**		**●**				10/11****
Royal College of Radiologists [195]				**●**	**●**						4/6^§§^
Piper [46]		**●**	**●**	**●**	**●**	**●**	**●**	**●**	**●**		10/10**
Royal College of Radiologists [196]				**●**	**●**					**●**	8/8*
NHS England [197]			**●**		**●**						4/6^§§^
Society of Radiographers [198]		**●**		**●**	**●**						7/8*
Royal College of Radiologists [199]	**●**										4/6^§§^
Snaith, Hardy, Lewis [200]	**●**	**●**		**●**	**●**						7/8*
Barter [201]			**●**	**●**		**●**			**●**		5/11^§^
Independent Cancer Taskforce [202]				**●**	**●**						5/6^§§^
Royal College of Radiologists [203]				**●**						**●**	8/8*
Royal College of Radiologists [204]	**●**					**●**					5/6^§§^
Royal College of Radiologists [205]	**●**									**●**	5/6^§§^
Booth, Henwood, Miller [206]				**●**	**●**						9/10^‡^
Hardy, et al. [207]	**●**		**●**	**●**	**●**				**●**	**●**	11/11^§^
Royal College of Radiologists [208]	**●**			**●**	**●**	**●**					5/6^§§^
Milner, Culpan, Snaith [209]			**●**	**●**			**●**	**●**			7/8*
Nightingale, McNulty [210]	**●**		**●**	**●**	**●**		**●**	**●**			6/6^§§^
Royal College of Radiologists [211]		**●**		**●**	**●**					**●**	8/8*
Snaith, Milner, Harris [212]				**●**			**●**		**●**	**●**	9/11****
Royal College of Radiologists [213]	**●**		**●**								4/6^§§^
Society of Radiographers [214]		**●**		**●**	**●**						7/8*
NHS England [215]	**●**			**●**	**●**						7/8*
NHS England [216]	**●**		**●**	**●**	**●**		**●**		**●**	**●**	5/6^§§^
Milner, Snaith [217]	**●**			**●**	**●**				**●**		8/8*
Lockwood [218]	**●**		**●**	**●**	**●**	**●**	**●**		**●**		8/8*
Benwell, Fowler [219]	**●**		**●**	**●**	**●**	**●**			**●**		7/8*
British Institute of Radiology [220]	**●**	**●**		**●**	**●**						5/6^§§^
Health Education England [221]	**●**	**●**	**●**	**●**	**●**					**●**	4/6^§§^
Society of Radiographers [222]		**●**		**●**	**●**		**●**				4/6^§§^
Society of Radiographers [223]		**●**		**●**	**●**						7/8*
Synergy News [224]		**●**		**●**	**●**		**●**				4/6^§§^
Kerrney [225]		**●**		**●**	**●**		**●**				5/6^§§^
Royal College of Radiologists [226]	**●**	**●**		**●**	**●**					**●**	8/8*
NHS England [227]	**●**	**●**	**●**	**●**	**●**		**●**		**●**	**●**	5/6^§§^
NHS England [228]	**●**		**●**	**●**	**●**						8/8*
Thom [229]	**●**			**●**	**●**	**●**	**●**	**●**	**●**	**●**	7/11^§^
Synergy News [230]	**●**		**●**	**●**	**●**		**●**				5/6^§§^
Royal College of Radiologists [231]					**●**	**●**			**●**		5/6^§§^
NHS England [232]	**●**		**●**	**●**	**●**		**●**	**●**	**●**		5/6^§§^
Care Quality Commission [233]	**●**	**●**	**●**	**●**	**●**		**●**			**●**	8/8*
Harcus, Snaith [234]		**●**		**●**							8/10***
Royal College of Radiologists [235]	**●**	**●**		**●**	**●**					**●**	8/8*
Society of Radiographers [236]		**●**		**●**	**●**						7/8*
NHS England [237]	**●**		**●**		**●**						5/6^§§^
Snaith, et al. [238]			**●**	**●**	**●**	**●**	**●**		**●**		7/8*
Royal College of Radiologists [239]	**●**	**●**		**●**	**●**					**●**	8/8*
Culpan, et al. [240]	**●**	**●**	**●**	**●**	**●**	**●**	**●**	**●**	**●**	**●**	7/11^§^
Society of Radiographers [241]		**●**		**●**	**●**						7/8*
Royal College of Radiologists [242]	**●**		**●**	**●**	**●**						5/6^§§^
Society of Radiographers [243]	**●**	**●**		**●**	**●**						5/6^§§^
NHS England [244]				**●**	**●**						5/6^§§^
Health Education England [245]		**●**	**●**		**●**						4/6^§§^
NHS England [246]		**●**	**●**		**●**						4/6^§§^
Health Education England [247]		**●**	**●**		**●**						4/6^§§^
Health Education England [248]		**●**	**●**		**●**						4/6^§§^
Stevens [249]	**●**		**●**	**●**	**●**	**●**	**●**	**●**	**●**		7/8*
Cuthbertson [250]		**●**		**●**	**●**	**●**	**●**	**●**			9/10^‡^
Harcus, Snaith [251]		**●**			**●**		**●**	**●**			7/10^‡^
NHS England [252]	**●**	**●**		**●**							5/6^§§^
Cuthbertson [253]	**●**			**●**		**●**	**●**			**●**	9/10^‡^
Royal College of Radiologists [254]	**●**	**●**		**●**	**●**					**●**	8/8*
Society of Radiographers [255]	**●**			**●**			**●**	**●**			4/6^§§^
Society of Radiographers [256]		**●**		**●**	**●**						7/8*
NHS England [257]		**●**		**●**							5/6^§§^
Price, Paterson [6]			**●**	**●**							6/6^§§^
Woznitza, et al. [258]	**●**	**●**		**●**		**●**	**●**	**●**			6/6^§§^
NHS England [259]		**●**	**●**	**●**	**●**						5/6^§§^
NHS England [260]	**●**	**●**	**●**	**●**	**●**	**●**	**●**	**●**	**●**		6/6^§§^
Milner, Barlow [261]	**●**		**●**	**●**	**●**		**●**		**●**		7/8*
Woznitza, et al. [262]				**●**		**●**	**●**	**●**			7/8*
Society of Radiographers [263]		**●**		**●**	**●**						7/8*
Society of Radiographers [264]		**●**		**●**							5/6^§§^
Royal College of Radiologists [265]			**●**	**●**	**●**						5/6^§§^
Royal College of Radiologists [266]	**●**	**●**	**●**	**●**	**●**					**●**	8/8*
Heales, Mills, Ladd [267]	**●**		**●**	**●**		**●**				**●**	4/11^§^
Society of Radiographers [268]				**●**		**●**					7/8*
Parliamentary Ombudsman [269]			**●**			**●**		**●**			5/6^§§^
Health Education England [270]		●		**●**							4/6^§§^
Akpan, Kitundu, Ekpo [271]			**●**						**●**		9/11^§^
College of Radiographers [272]	**●**			**●**		**●**					5/6^§§^
Government Select Committee [273]	**●**	**●**		**●**	**●**					**●**	5/6^§§^
Cain, et al. [51]	**●**		**●**	**●**		**●**	**●**	**●**	**●**	10/10**	Cain, et al. [51]
Wood [274]	**●**	**●**		**●**	**●**		**●**	**●**		**●**	6/11^§^
Shepherd, Lourida, Meertens [57]			**●**	**●**	**●**		**●**	**●**	**●**	**●**	8/11^§^
NHS England [275]	**●**	**●**	**●**	**●**	**●**				**●**		5/6^§§^
Royal College of Radiologists [276]	**●**	**●**								**●**	8/8*
Royal College of Radiologists [277]		**●**		**●**		**●**	**●**	**●**			5/6^§§^
Health Education England [278]		**●**		**●**	**●**						4/6^§§^
Sevens, McGivern [279]		**●**	**●**	**●**	**●**				**●**		7/8*
College of Radiographers [280]		**●**		**●**	**●**						7/8*
The Kings Fund [281]		**●**	**●**								6/6^§§^
Academy of Medical Royal Colleges [282]				**●**	**●**	**●**					5/6^§§^
Murphy, Nightingale, Calder [283]		**●**		**●**	**●**	**●**	**●**	**●**			8/11^§^
Murphy, Nightingale, Calder [284]		**●**		**●**	**●**	**●**	**●**	**●**		**●**	11/11^§^

**Fig. 2:**
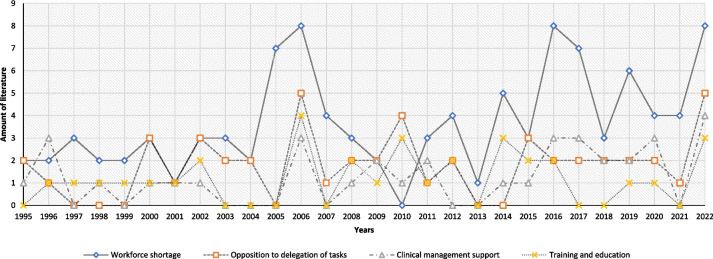
Data analysis of patterns and trends of barriers in the found literature

The breakdown of literature was predominantly from the micro level (*n* = 126; 52.2%), with lesser evidence from meso (*n* = 63; 26.1%) and macro levels (*n* = 52; 21.6%). Data analysis and synthesis of the empirical evidence examining the barriers to implementing the reporting radiographer service (Fig. 2) highlighted patterns and trends in publications over four main themes. Workforce shortages [29, 108, 136, 176, 195, 196, 199, 203, 211, 226, 235, 239, 254, 265, 266, 273, 276, 285] (*n* = 19/28 years) were the leading theme between 1995 and 2022. The barriers included examples of the limited number of consultant radiologists within England [108, 136, 176, 184, 185, 196, 203, 211, 226, 235, 239, 242, 254, 266, 273, 276, 285], due to variables of training numbers, current workforce, and expectations of retirement of staff, which consequentially influenced professional body (meso-level) preferences of outsourcing or regional radiologist networking [108, 173, 181, 184, 195, 196, 199, 203, 211, 213, 226, 235, 239, 242, 254, 265, 266, 276, 285] as opposed to supporting (micro-level) internal skills mix working in departments. Conversely, the limited consultant radiologists workforce affected the availability (micro and meso-level) for mentoring and supporting radiographers in reporting education and training programmes [6, 12, 13, 80, 81, 88, 104, 153, 162, 163, 174, 200, 209, 218, 219, 225, 229, 240, 250, 253, 279, 283, 284]. Reciprocally the limited radiographer workforce also limits the availability of clinical departments (micro-level) to release radiographers to attend educational programmes and support the release of staff for advanced practice roles [200, 209, 218, 219, 250, 253, 283, 284]. Furthermore, there are trends that link workforce limitations with radiologists' opposition (micro and meso-level) to the delegation of tasks [6, 12, 13, 73, 76, 78, 80, 81, 88, 104, 108, 114, 116, 117, 120, 122, 132, 133, 138, 144, 147, 158, 162, 164, 166, 167, 169, 173, 179, 181, 200, 201, 204, 205, 209, 219, 225, 229, 231, 253, 261, 265, 274, 276, 283, 284, 286] (Fig. 2), and to a minor degree, management support (micro-level) for radiographer reporting training [163, 174, 200, 209, 218, 219, 225, 229, 240, 250, 279, 283, 284]. It is noted there was a trend (micro and meso-level) in the literature debating the training standard and curricula [13, 81, 83, 88, 93, 104, 108, 114, 133, 155, 158, 167, 185, 284] between radiologists and reporting radiographers, often centred around medical and non-medical perspectives that were often used to support barriers to adoption of the service delivery.

**Fig. 3:**
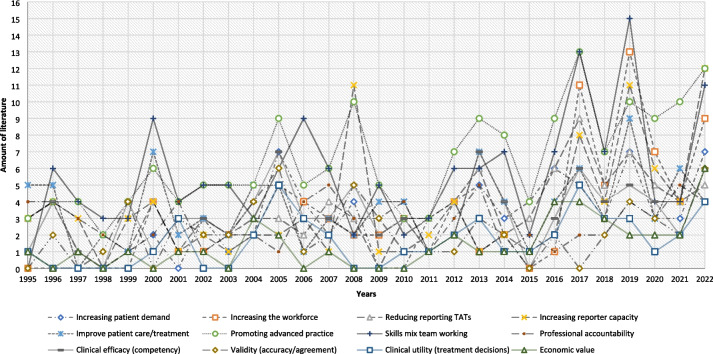
Data analysis of patterns and trends of enablers in the found literature

Exploring the enabler data by theme (Fig. 3) demonstrated twelve themes with promoting advanced practice [6, 13, 26, 38–41, 45–47, 49, 51–59, 73–75, 77, 79, 80, 83–86, 88–91, 94, 96, 99, 101–107, 110, 111, 113, 115–124, 127, 128, 130, 134, 138, 139, 141, 142, 144, 146–148, 150, 151, 154–159, 161–166, 170, 171, 174–178, 180–182, 185, 187, 188, 190–196, 198, 200–203, 206–210, 212, 214, 215, 217–230, 232–236, 238–241, 243, 245, 247–250, 253–261, 263–265, 268, 270, 273–275, 277–280, 282–285, 287, 288] in radiographer reporting (*n* = 19/28 years) supported at macro, meso and micro-levels as the leading theme between 1995 and 2022, closely followed by skills mix working (*n* = 14/28 years) in Fig. 3. There were trends related to specific peaks of literature over the years which link macro-level governmental NHS reform policy in 2000 to remove "*traditional and unnecessary demarcations and introduce more flexible working practices*" [95–98, 100, 101], the 2006 push to increase the advanced practice workforce numbers [134, 135], the 2012 policies advocating reporting radiographers to speed up reporting Turnaround Times (TATs) [177, 178], the 2014 five year forward [197], the 2017 Cancer Workforce plan [227, 287, 289], and the 2019 NHS long term plan [237, 244, 246, 247, 252], and the Richards [259] and the Getting it Right First Time [260] reports to improve patient care with increasing the workforce and reporter capacity, supporting other enablers such as promoting advanced practice and skills mix to achieve those targets (Fig. 3). Backed by meso-level professional policy guidance and statements in 1997 [85] of the rationale of the role, 2006 defence of the role [138], with 2007 interprofessional team working agreements [142], 2010 defining terminology and roles [165, 166], 2012 further team working endorsement [182, 183], 2013 formalising roles [188], acceptance of roles [208] and quality standards [272] in reporting and training [277].

## Discussion

The main enabler themes (*n* = 12; Fig. 3) that have influenced and assisted facilitation of the radiographer X-ray reporting role and the barrier themes (*n* = 4; Fig. 2) that have impacted, restricted and impeded the implementation and its progression can be explored using Kingdon’s [290, 291] Multiple Streams Framework (MSF) to explore the different rational solutions that exist and change overtime to any issue. Kingdon’s [290] uses the theoretical MSF to trace how the different macro, meso, and micro-levels (classed as streams in the MSF [290]) interact and cross-over to influence policy agendas, and how coupling of different streams (macro, meso, and micro-levels) can influence solutions through connecting to build flexibility and a momentum of change (historical, socio, political, organisational, geographical, governance and resource factors).

### Context

The historical context of demand of patient imaging referrals [29] against the capacity of the workforce to perform the reporting of X-ray imaging examinations has been at the forefront of the literature [96, 108, 134, 176, 178, 181, 185, 196, 203, 211, 220, 226, 227, 235, 239, 240, 247, 254, 266, 276, 292], and the contemporary [293] perspective shows no signs of abating. This unequilibrium of streams in NHS service delivery has been and still is the primary context to this advanced practice, with patients being at the heart of everything that is done in the NHS [294]. To address these problems, there has been what Kingdon [290] would describe as ‘policy windows’ at the macro-level [245, 247, 248, 278, 292] of short-term funding policy agendas to increase training of reporting radiographers, although sustained annual investment in the long term is required to sustain the reporting radiographer workforce.

There was a notable lack of patient and public involvement (PPI) and contribution in the research and evidence surrounding radiology reporting and the reporting radiographer role. Specifically from active involvement as either advising, co-designing, data collection or, of provision of first-hand experience of the service in case studies and reports. The inclusion of PPI perspectives of reporting delays, workforce shortages, and skills mix working may provide valuable insight to factors that shape the service that have not be identified from the existing literature.

### Culture

Assumptions and attitudes without evidence have historically inhibited professional culture (meso-level) from adopting interdisciplinary skills mix roles that overlap traditional boundaries, and instead preference monopolies and turfs [295] (meso-level professional bodies [108, 195, 213, 286]) with less acceptance of collaboration or acceptance of individual qualifications, abilities and competencies that enhance patient outcomes. One such historical argument was the 'gold standard' [296] of reporting, a historical medical term applied by radiologists to describe their performance ability in reporting. Although, based primarily on opinion with little evidence of the rigorous threshold of accuracy beyond training assessment [297], which was seemingly at odds with the threshold of clinical error reported [298–300]. Current literature terminology now refer to terms ‘reference standard’ which can be applied to any profession reporting, or ‘ground truth’ collaborated by multi-professional diagnostic tests (blood reports, histology results, surgical findings, etc.). Arguments and debates around reporting accuracy and abilities for medical (radiologist) versus non-medical (radiographer) training have now subsided with interprofessional body consensus and acceptance (Kingdon’s policy window [290]) of radiographer reporting training and competency [142, 182, 272, 277]. Supported by evidence of radiographers reporting all patient groups, ages and referral pathways to fully justify the role [200, 218, 219].

The clinical experience within the literature to support the adoption of reporting radiographers can be reflected in the combination of multiple streams [290] of macro [93, 118], meso [74, 108, 142, 180, 189, 196, 198, 203, 211, 214, 220, 223, 224, 226, 235, 236, 239, 243, 254–256, 263, 266, 268, 276, 285], and micro-levels through surveys, case studies, and commentaries [86, 104, 111, 144, 194, 249] providing anecdotal reflection and consensus as to the socio, political, and historical impact and importance of embedding the policy agenda of reporting radiographers in healthcare practice to improve local service delivery.

### Environment

To implement sustainable adoption of the role nationally has required substantial research to ascertain its value against the environmental backdrop of annual reduced fiscal investment [301] in NHS healthcare services. The evidence (micro-level) to support the advanced practice education and training [38, 42, 46, 158, 174, 234, 251, 277, 279, 284, 302], and the efficacy and ability of radiographers in the role to perform to high standards has been well conceived (Table 6) and designed [37, 39, 105, 106, 112, 120, 303–306], assessed [38, 43, 44, 46–48, 51, 54, 58, 107, 151, 175], and its associated downstream impact on cost [58, 59, 220] and patient treatment and management [53–57, 191, 201, 207, 307] has been critical to the success of the national roll-out and implementation of the role since 1995.

Of note within the literature there is evidence of variance and influence from the different macro, meso, and micro-levels [290] to the uptake and implementation between geographic regions throughout England [111, 164, 200, 209, 217, 249, 262, 279, 283] potentially due to regional access to training programmes [13, 46, 86, 91, 104, 174, 234, 251, 279], and funding [131, 134, 135, 145, 176, 221, 245, 247, 248, 270, 278, 292]. Additionally, the progression of image acquisition technology and display equipment has helped to progress the role.

The move from daylight processing of X-ray hard-copy film in 1995 to contemporary Computed Radiography (CR) and Digital Radiography (DR) systems with storage and display of images on picture archiving and communications systems (PACS) has revolutionised the image quality for reporting subtle findings. Moreover, this has impacted the manner in which reporting sessions now occur [308], from individual radiology department reporting offices in 1995 using light boxes to display individual examinations to modern twenty-first century reporting computer monitors. The role of reporting has adapted to include both on-site (hospital) reporting stations and off-site remote home reporting stations [309] that increase the ability for staff to participate in out of hours (insourcing) reporting which may be beneficial to reduce backlogs.

Furthermore there is a growing body of literature debating and discussing the trialling Artificial Intelligence (AI) software at different macro, meso, and micro-levels [290] to assist and support in the automation of some tasks in the chain of reporting of X-ray examinations [310]. Although, notable advances and trends in the use of AI have been identified [311, 312], the safe integration of AI is as yet more of a second reader assistance and decision support [313] than replacement of radiologists and reporting radiographers.

### Leadership

Receptiveness for change borne by governmental agendas and policymaking [290] (macro-level) [172, 197, 216, 227, 232, 233, 237, 244, 246, 252, 259, 260, 287, 314] to improve healthcare services and delivery for modern society, has, at times, encountered meso-level opposition [76, 93, 108, 213]. But strong leadership at the meso-level [6, 74, 75, 117, 123, 162, 165, 315] have helped shape the succession planning and sustainability of the radiographer reporting role over the years to counter alternatives such as outsourcing reporting backlogs to private companies as a quick fix solution that wastes limited NHS finances that could be spent on increasing the reporting workforce capacity [108, 203, 213, 226, 235, 239, 254]. Future progression of the role requires combination [290] of meso-level professional body leadership to shape direction and inclusion within workforce planning to sustain macro-level governmental healthcare proposals to target healthcare priorities such as faster reporting TATs [233, 260], cancer diagnosis [172, 202, 240, 246, 247, 287] and community diagnostic hubs [233, 259, 260].

It was noted from the literature a limiting factor to monitoring the workforce shortages was a lack of verified and accurate data of how many reporting radiographers were embedded in roles within the NHS in England, as often not all NHS trusts returned data so an incomplete picture of the workforce exists [180, 189, 198, 214, 223, 236, 241, 256, 263] which is hinders decisive future workforce planning.

Additionally, considering the wider perspectives and implications of this skills mix practice. Reporting by radiographers is now established in UK clinical practice, and there is growing evidence of future global opportunities for implementing trained radiographer (often termed a radiologic technologist, or medical radiation technologist internationally) reporting in countries [316] with similar drivers around an increasingly unstable equilibrium of patient demand and reporting workforce supply. Already Australia [147], Canada [317], Denmark and Sweden [318], Ghana [319], Mexico [320], Nepal [321], Norway [322], South Africa [323], and Uganda [324] have made tentative steps in radiographer reporting trials to gauge stakeholder acceptance. However, it is noted the individual macro, meso and micro-level barriers and enablers for each country contain large socioeconomical, cultural, political, professional, and healthcare system differences that require exploring before the skills mix clinical practice of reporting by radiographers is fully adopted across each of these countries.

This study acknowledges some limitations in the methodology, specifically with regards to the search strategy used to identify relevant articles. The use of both broad and specific search terms was an attempt to minimise the risk of missing relevant publications, but it is possible that some pertinent articles may have been excluded or missed. This paper should not be considered an exhaustive list of all the publications in this field; but rather highlights some of the most influential papers to date. Likewise there is an acknowledgment of the limitations of quality in detail, transparency, rigour and evidence between professional and governmental policy, guidance, and statements, and clinical practice level studies and research. It's important to consider these limitations when interpreting the findings of the study.

## Conclusion

The literature since 1995 has provided a complex interplay of policy professional and practice streams which have been more or less aligned over the years. The literature has reframed the debates on implementation of the radiographer reporting role and has been instrumental in shaping clinical practice. There has been clear influence upon both meso (professional body organisations) and macro-level (governmental/health service) agendas, policies, and guidance that have shaped change at micro-level NHS Trust organisational levels. There is evidence of a shift in culturally intrenched legacy perspectives within and between different meso-level professional bodies around skills mix acceptance and role boundaries. This has helped shape capacity building of the reporting workforce and radiographer skills development.

The enabling evidence provides clarity and definition of the X-ray radiographer reporting role, and its efficacy, utility, and clinical validity, and is seen as beneficial to the healthcare service, particularly in light of mounting patient demand pressures. The enabling drivers found within the evidence included radiographers reporting all patient groups, ages and referral pathways to evidence the role beyond task dependent activities.

Nevertheless, some challenges and barriers at the meso and micro-level were identified, predominately due to professional body slowness to endorsing team working and implement skills mix roles. Workforce shortages remain a consistent barrier and limit the capacity of reporters (both radiology registrars and diagnostic radiographers). With funding and training numbers the main limiting factors halting future growth of the workforce to provide consistent reporting staff to address the increasing demand of patient referrals, which requires addressing at the macro national level to adequate address service delivery shortfalls.

Future work would do well to interweave the patient perspective of reporting delays, workforce and skills mix, which is currently lacking in the published literature. As well as census surveying of reporting radiographers employed within NHS Trusts in England to guide workforce planning and sustainability of the role to support macro-level governmental healthcare priorities.

### Supplementary Information

**Additional file 1.** PRISMA_2020_abstract checklist. Completed PRISMA checklist for abstract.

**Additional file 2.** PRISMA_2020_checklist. Completed PRISMA checklist for systematic review’s.

**Additional file 3.** Raw Data.

## Data Availability

All data generated or analysed during this study are included in this published article and its supplementary information files.

## Abbreviations

AI: Artificial Intelligence
BIR: British Institute of Radiology
BMA: British Medical Association
CoR: College of Radiographers
CR: Computed Radiography
DoH: Department of Health
DR: Digital Radiography
GMC: General Medical Council
JBI: Joanna Briggs Institute
NHS: National Healthcare System
MSF: Multiple Streams Framework
PACS: Picture archiving and communications systems
PICO: Population, Intervention, Comparison, Outcomes study design
PPI: Patient and public involvement
PRISMA: Preferred Reporting Items for Systematic Reviews and Meta-Analyses
PRISMA-P: Preferred Reporting Items for Systematic Reviews and Meta-Analyses Protocols
PROSPERO: Prospective Register of Systematic Reviews
RCR: Royal College of Radiologists
RCT: Randomised Control Trial
SCoR: Society and College of Radiographers
SOR: Society of Radiographers
TATs: Turnaround Times
UK: United Kingdom
US: Ultrasound
